# Possible Role of Accessory Proteins in the Viral Replication for the 20I/501Y.V1 (B.1.1.7) SARS CoV-2 Variant

**DOI:** 10.3390/pathogens10121586

**Published:** 2021-12-07

**Authors:** Dimpal A. Nyayanit, Prasad Sarkale, Anita Shete-Aich, Abhinendra Kumar, Savita Patil, Triparna Majumdar, Shrikant Baradkar, Pranita Gawande, Sreelekshmy Mohandas, Pragya D Yadav

**Affiliations:** Indian Council of Medical Research-National Institute of Virology, Pune 411021, India; nyayanit.dimpal@gmail.com (D.A.N.); prasadsarkale123@rediffmail.com (P.S.); anitaaich2008@gmail.com (A.S.-A.); abhinendra.biotech@gmail.com (A.K.); varshapatil111@yahoo.com (S.P.); triparna.majumdar@gmail.com (T.M.); sbaradkar3@gmail.com (S.B.); pranita1994gawande@gmail.com (P.G.); sreelekshmy88@gmail.com (S.M.)

**Keywords:** 20I/501Y.V1, SARS CoV-2, replication, RPKM, TCID_50_, next-generation sequencing

## Abstract

The emergence of new severe acute respiratory syndrome coronavirus-2 (SARS CoV-2) has been a global concern. The B.1.1.7 variant of SARS CoV-2 is reported to cause higher transmission. The study investigates the replication cycle and transcriptional pattern of the B.1.1.7 to hypothesis the possible role of different genes in viral replication. It was observed that the B.1.1.7 variant required a longer maturation time. The transcriptional response demonstrated higher expression of ORF6 and ORF8 compared to nucleocapsid transcript till the eclipse period which might influence higher viral replication. The number of infectious viruses titer is higher in the B.1.1.7, despite a lesser copy number than B.1, indicating higher transmissibility. The experimental evidence published linked ORF6 and ORF8 to play important role in replication and we also observed their higher expression. This leads us to hypothesis the possible role of ORF6 and ORF8 in B.1.1.7 higher replication which causes higher transmission.

## 1. Introduction

The recent identification of the new Severe Acute Respiratory Syndrome Coronavirus-2 (SARS CoV-2) variants has been of public health concern globally, after its first report from China in December 2019 [1]. SARS CoV-2 encodes for structural [E (envelope), N (nucleocapsid), M (membrane), and S (spike)], non-structural proteins and six accessory proteins (ORFs 3a, 6, 7a, 7b, 8, 10) [2]. The expression of these proteins is responsible for the replication of the SARS CoV-2with its hosts.

Information on the growth kinetics helps in understanding the virus-host interaction. It is also known that like other RNA viruses, the mutation rate of SARS CoV-2 is higher in comparison to other virus groups [3]. The growth kinetic study of the SARS-CoV demonstrated the viral release at 7th-hour post-infection (hpi) in the Vero-E6 cells [4]. The growth kinetics study for the SARS CoV-2 demonstrates a similar trend to SARS-CoV, where the virus was observed in the supernatant from 7th hpi [5].Further, limited data is present on the transcriptional response of the SARS CoV-2 proteins after it infects a host cell [5,6,7,8] at different time-points. Comparison of respiratory viruses with the SARS CoV-2 in cell lines and animal systems revealed a reduced cytokine and Interferon (Type I and III) response [6].

Transcriptome analysis can be used for general expression analysis of the cellular system. This system can be further modified to understand gene expression under specific conditions or at particular time [9,10]. Targeted sequencing was used to identify relevant pharmacogenes to facilitate precise medicine [11]and single-cell analysis was used to analyze allele specific gene expression different developmental stages in mouse embryo [12]. Further transcriptome network analysis has also been used to understand the severity of asthmatic condition in children [13]. Transcriptome comparison of the SARS-CoV, MERS-CoV and the SARS CoV-2 showed the suppression of the mitochondrial pathway and the glutathione metabolism [7]. Time-point analysis by Sun et al demonstrated early host response to the SARS CoV-2entry to the host cell in comparison to SARS-CoV and MERS-CoV [8].

Delayed appearance of the cytopathic effect for the B.1.1.7 variant, with similar passage history, in comparison to the B.1 variant prompted us to investigate further. This study was carried out to understand the time required to complete the replication cycle of the B.1.1.7, a variant of concern (VOC). Further, in vitro transcriptional response generated at various time points was assessed.

## 2. Materials and Methods

The present study had approval by the Institutional Animal Ethics and Biosafety Committee of Indian Council of Medical Research (ICMR)—National Institute of Virology (NIV), Pune. This study is based on the expression of virus in a cell culture and does not include any experimental animal or human models.

The 20I/501Y.V1 (B.1.1.7) SARS-CoV-2 isolate used in this study from the throat/nasal swab of United Kingdom traveler to India and submitted to GISAID (GISAID Number: EPI_ISL_825086) during late December 2020 [14]. Vero CCL81 grown in the 24-well plate was infected with the 20I/501Y.V1 SARS CoV-2 strain (TCID_50_:10^5.5^/mL, Vero CCL81 P-3). A 0.1 multiplicity of infection (MOI) was used to study the viral replication and the transcriptional response of the host Vero CCL81 cell line in a triplicate set. Cell pellet and supernatant were harvested at the defined time intervals (0, 30 min, 1 h, 2 h, 4 h, 6 h, 7 h, 8 h, 9 h, 10 h, 12 h, 24 h, 48 h, 72 h, 96 h, 120 h and 144 h) and stored at −80 °C till further use. TCID_50_ and Cyclic threshold (Ct) value for the *E* gene were determined calculated for both the supernatants and cell pellets using the end-point titration assay and real-time PCR method [15].

Vero CCL81 cells were infected with the B.1 strain (GISAID number: EPI_ISL_420545; TCID_50_: 10^6.5^/mL, Vero CCL-81 P-3) and B.1.1.7 strain (TCID_50_: 10^5.5^/mL, Vero CCL81 P-3) in a 24 plate well. The cell supernatant was harvested on the 3rd and the 4th PID for both the strains and stored at −80°C till further use. TCID_50_ and Ct value (E gene) was determined using the end-point titration assay and real-time PCR method[15] for both the strains. 

RNA was extracted from cell pellet and supernatant harvested at different time intervals. cDNA libraries were prepared for the extracted RNA and used for library preparation using the Illumina TruSeq Stranded mRNA LT Sample Preparation Kit. The libraries were sequenced using the Illumina platform. The total viral reads generated for the libraries of the harvested cell pellets and supernatants were mapped with the reference genome (accession number: NC_045512.1). The in vitro response for each gene transcript was quantified using the reads per kilobase million (RPKM) methods which normalizes the reads against the sequencing depth and gene length. The RPKM values were calculated using the CLC Genomics Workbench software (version 20.0.4, Qiagen Bioinformatics) for each time point. Further, the average of the observed RPKM values of each SARS CoV-2 (20I/501Y.V1) genes transcripts was normalized against the nucleocapsid transcript at the respective time point.

## 3. Results and Discussion

### 3.1. 20I/501Y.V1 (B 1.1.7) SARS- CoV- 2 Variant Is a Slow-Growing Virus

**Figure 1:**
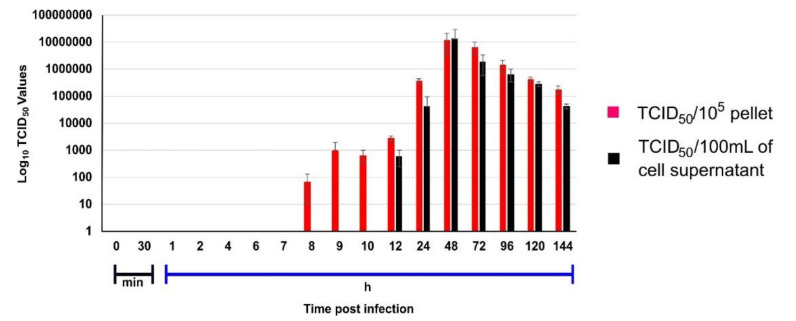
Growth kinetics of SARS CoV-2 (20I/501Y.V1) in Vero CCL81 cell: Vero CCL81 cells were infected with 20I/501Y.V1 (0.1 MOI of 10^6^/mL TCID_50_) in triplicate sets. The cell pellet and supernatant were harvested at defined time intervals and viral titers were estimated using endpoint titration assay in Vero CCL81 cells. TCID_50_ of virus present within the cell pellet and in the supernatant is indicated with red and black colour respectively. Error bars depict the standard deviation observed in a triplicate set.

The intracellular live virus in pellet and extracellular live virus in supernatant was calculated in a triplicate set using endpoint titration assay in Vero CCL81 cells at defined time intervals. The presence of live virus was detected in pellet beginning from the 8 hour’s post-infection (hpi) while the virus was released in the supernatant only at 12th hpi (Figure 1). The time lag of 3 h till the first virus appearance in the supernatant was observed. In the case of the B.1 variant, the virus was observed in the supernatant approximately within an hour after its appearance in the cell pellet [5]. The delayed observance of the B.1.1.7 in the supernatant in respect to B.1 led us to interpret that B.1.1.7 has a slow growth cycle caused due to its delayed maturation time. 

### 3.2. Higher Expression of Accessory Transcripts Possibly Leads to an Increase in Viral Replication

**Figure 2:**
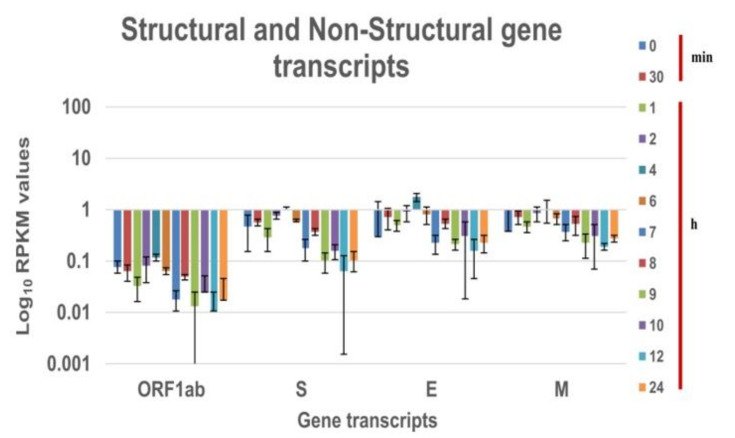
Expression of SARS CoV-2 (20I/501Y.V1) structural and non-structural transcripts in Vero CCL-81 at different time intervals in cell pellet: Normalized RPKM values of the SARS CoV-2 (20I/501Y.V1) genes from the cell pellet of Vero CCL-81 at different time intervals. The RPKM values are normalized with the nucleoprotein transcript values at the respective time intervals. Log10 RPKM values for the genes are plotted on the X-axis and gene transcripts on the Y-axis. Different colours are the log 10 RPKM values at that time intervals Average RPKM value is plotted and the standard deviation is shown in the error bar.

**Figure 3:**
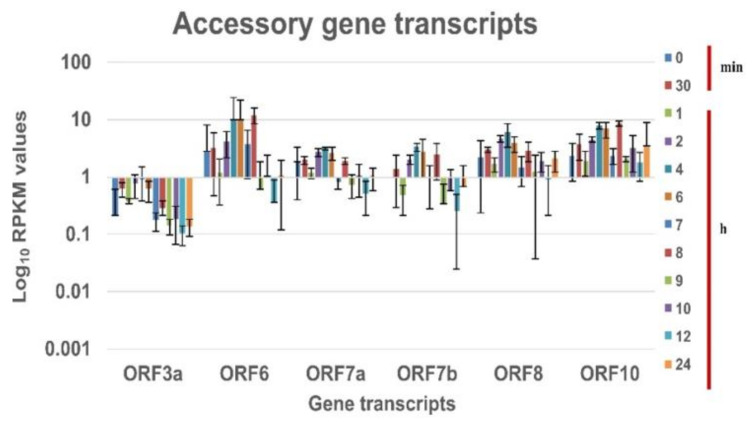
Expression of SARS CoV-2 (20I/501Y.V1) accessory protein transcript in Vero CCL-81 at different time intervals in cell pellet: Normalized RPKM values of the SARS CoV-2 (20I/501Y.V1) genes from the cell pellet of Vero CCL-81 at different time intervals. The RPKM values are normalized with the nucleoprotein transcript values at the respective time intervals. Log10 RPKM values for the genes are plotted on the X-axis and gene transcripts on the Y-axis. Different colours are the log 10 RPKM values at that time intervals Average RPKM value is plotted and the standard deviation is shown in the error bar.

In vitro transcriptional response for the SARS CoV-2 genes was obtained using the next-generation sequencing of the cell pellet harvested at defined time intervals for the B.1.1.7 variant. The nucleocapsid transcript is expressed at a higher level in comparison to other viral transcripts at all-time points of experiments for SARS CoV-2 [5,16]. The RPKM values observed was hence normalized with the nucleocapsid expression. Figure 2 and Figure 3 is the log10 plot of the normalized RPKM values obtained for structural protein transcripts & non-structural polypeptide transcripts and accessory protein transcripts respectively at a defined time interval. The structural, non-structural, and accessory protein transcripts of the B.1 virus were always expressed in lower levels as compared to nucleocapsid transcript [5]. However, in the case of the B.1.1.7 variant, a few of the structural and accessory protein transcripts are expressed more than the nucleocapsid protein transcript. The structural gene transcripts of B.1.1.7 are expressed in lower levels as compared to the nucleocapsid transcript at the time points (Figure 2). But, the accessory protein ORF8 and ORF10 transcripts are expressed in higher levels at every time point in the study. The ORF6, ORF7a and 7b transcripts are expressed at higher levels till the eclipse period of the virus and drops later. The expression of the ORF3a transcript is at lower levels for each time point (Figure 3). 

It has been reported earlier that pathogenic viruses increase viral replication by antagonizing the nuclear import to suppress the host immune response [17]. ORF6 protein of the SARS CoV-2 bind to the Nup98-Rae1 thereby targeting nuclear import [18], indicated by its higher expression levels. Further, the role of ORF8 in viral replication was also reported [19]. This indicates that the higher level of ORF6 and ORF8 can be associated with higher viral replication. With a similar observation in the increased expression level of the ORF6 and ORF8 transcripts, it could be hypothesized that B.1.1.7 virus has higher viral replication. 

### 3.3. Higher Transmission of B.1.1.7 Variant Can Be Possibly Liked to Larger Infectious Virus Titer

Total destruction in the cell culture monolayer for B.1 and B.1.1.7 was observed on the 3rd PID and 4th PID respectively, hence the sample for the B.1.1.7 was harvested on the 4th-day -post-inoculation (PID) whereas for B.1 it was the 3rd PID. Further a variation in the infectious virus titer was observed on the first detection in the supernatant for both B.1.1.7 variant (~1 × 10^2.8^ per mL at 12 hpi ) and B.1 variant (~1 × 10^1.5^ per mL viral titer at 8 hpi). To understand the difference of infectious virus titre we infected both the strains to the Vero CCL81 cells and retrieved tissue culture fluids for the 3rd and 4th PID. It was observed that the infectious virus titer for B.1 variant was reduced by 30.8% on the 4th PID (3rd PID: 4.57 × 10^5^; 4th PID: 3.16 × 10^5^). However, in the case of the B.1.1.7 variant, the infectious virus titre was reduced by 74.8% on the 4th PID (3rd PID: 3.98 × 10^6^; 4th PID: 1.0 × 10^6^). This indicated that the amount of the infectious particle was less at the 4th PID for B.1.1.7 variant in comparison to B.1 variant and the observed ratio for B.1:B.1.1.7 is 69.2:25.2 (2.75:1).

Further, the viral load of the B.1.1.7 variant was 7.86 × 10^11^ copies/mL at 4th PID in comparison to the 3rd 5.36 × 10^11^ copies/mL which is higher. An increase of ~1.46 fold in the viral load was observed at the 4th PID in comparison despite a 74.8% reduction in infectious virus particles reduced by titer. On the other hand,B.1 variant had ~1.35 fold increase in viral load but, the infectious virus particles reduced only by 30.8% indicating a higher contribution of the live virus [3rd PID: 1.69 × 10^12^ copies/mL and 4th PID: 2.29 × 10^12^ copies/mL]. The real-time data indicates the B.1 variant to have a higher viral copy number. Overall higher viral copy number and a lesser reduction in viral titer were observed for the B.1 variant. The viral titer of the B.1.1.7 variant drops at a higher rate in the 3rd and the 4th consecutive days however the amount of the infectious virus is still larger in comparison to the B.1 variant indicating replication. This makes it highly transmissible leading to a higher number of COVID-19 cases [20] but having reduced pathogenicity due to lesser infectious viral particles.

## 4. Conclusions

This study reveals that B.1.1.7 SARS CoV-2 variant is a slow-growing virus. Further, a higher replication along with reduced infectious viral titer was hypothesized to be linked to higher transmission with reduced pathogenicity.
